# Differential plasma proteome analysis in patients with high-altitude pulmonary edema at the acute and recovery phases

**DOI:** 10.3892/etm.2014.1548

**Published:** 2014-02-14

**Authors:** YINGZHONG YANG, LAN MA, WEI GUAN, YAPING WANG, YANG DU, QIN GA, RI-LI GE

**Affiliations:** 1 Research Center for High Altitude Medical Sciences, Qinghai University School of Medicine, Xining, Qinghai 810001, P.R. China; 2 Department of Respiratory Medicine, Qinghai University Affiliated Hospital, Xining, Qinghai 810001, P.R. China

**Keywords:** high-altitude pulmonary edema, plasma, proteome, two-dimensional gel electrophoresis, apolipoprotein

## Abstract

This study aimed to investigate the differential expression of plasma proteins in patients suffering from high-altitude pulmonary edema (HAPE) at different phases. A complete proteomic analysis was performed using two-dimensional gel electrophoresis followed by mass spectrometry in three patients with HAPE at the acute stage and recovery phase. Comparisons between the expression patterns of the patients with HAPE at the two different phases led to the identification of eight protein spots with a >1.5-fold difference in expression between the acute and recovery phases. These differentially expressed proteins were apolipoproteins, serum amyloid P component, complement components and others. Apolipoprotein A-I (Apo A-I), serum amyloid P component and fibrinogen were overexpressed in the patients with HAPE in the acute stage compared with their expression levels in the recovery phase. However, Apo A-IV and antithrombin-III were overexpressed in the patients with HAPE in the recovery phase compared with their expression levels in the acute stage. The results indicate that the differential plasma proteome in patients with HAPE may be associated with the occurrence of HAPE, and the expression changes of Apo A-I and A-IV may offer further understanding of HAPE to aid its prognosis, diagnosis and treatment.

## Introduction

High-altitude pulmonary edema (HAPE) is a life-threatening respiratory disease that occurs in previously healthy people within 2–4 days of rapid ascent and exposure to an altitude >2,500 m above sea level (1–3). HAPE is a noncardiogenic pulmonary edema characterized by high pressure in the pulmonary arteries, edema in pulmonary interstitial tissues and alveoli and non-uniform pulmonary vasoconstriction, which results in pulmonary capillary stress failure and a high permeability type of edema (4,5). Although the hypobaric hypoxia is a major trigger factor, the exact mechanism underlying the development of HAPE remains unclear.

Previous studies have observed high levels of inflammatory cells in the bronchoalveolar lavage fluid of patients with HAPE (6–9). A marked increase in total cells, with macrophages being predominant, along with elevated levels of cytokines, including interleukin (IL)-6, IL-8 and tumor necrosis factor-α has been identified (9). Previous studies have identified changes in the levels of various cytokines in the plasma of patients with HAPE, but have not shown complete expression profiles (8–13). Recently, proteomic analyses have greatly facilitated the comprehensive cataloging of protein expression profiles, in not only cell lines but also in clinical samples, including serum/plasma, urine, spinal fluid, synovial fluid and tissues (14). Certain scholars have focused on determining the biomarkers in the plasma of patients with HAPE (19–17). Proteomics is one of the most powerful biological research techniques and has the potential to simultaneously detect differentially expressed proteins (18).

In the present study, two-dimensional gel electrophoresis (2-DE) was used to observe the plasma proteome profile in the acute stage and the recovery phase in the same patients with HAPE. The resulting gels were then compared to identify proteins that were differentially expressed and the proteins were determined by mass spectrometry. The comparison of proteomic results between the acute stage and the recovery phase was conducted to improve understanding of the pathogenic mechanisms of HAPE and the developmental changes in the plasma proteome of patients with HAPE during transition from the acute stage to the recovery phase.

## Material and methods

### Patients

All patients with HAPE (n=20) had been hospitalized in Yushu People’s Hospital (Yushu, China) between March and June 2012 due to the onset of HAPE within 1–3 days of arrival at Yushu (3,760 m above sea level). The patients had arrived at the Yushu area as construction workers following an earthquake (magnitude, 7.1) on April 4, 2010. The diagnosis of HAPE was based on chest X-rays and standard diagnostic criteria, which was in accordance with the Lake Louise scoring system (19). The patients met the criteria for HAPE at the onset of the disorder and recovered promptly with hospitalization. Examinations to exclude any additional diseases were conducted in the hospital after their recovery. The patients with HAPE were all Han-Chinese males who had been born and resided at lowland levels. These individuals were healthy and had previously shown no signs of sickness. A total of 20 highland healthy Han-Chinese controls who were resistant to HAPE (HAPE-r), were randomly selected from the coworkers of the patients with HAPE, matching the patients in age, gender, ethnicity and working conditions. These subjects remained healthy after working at Yushu for ≥3 months, without suffering from HAPE or high altitude cerebral edema. As lowland controls, 20 healthy Han-Chinese individuals from Xining, China (2,260 m above sea level) were randomly selected, matching the other two groups in age, gender, ethnicity and working conditions. With informed consent from all participants, 5 ml venous blood samples (with EDTA-Na as an anticoagulant) were collected from each patient with HAPE in the acute stage prior to treatment and in the recovery phase following the termination of treatment. The whole blood was immediately separated to blood cells and plasma by centrifuging at 1,500 × g for 10 min and the plasma was stored in liquid nitrogen. The plasma from three of the patients with HAPE in the acute stage and recovery phase was processed by 2-DE differential display with linear immobilized pH gradient (IPG) strips of 17 cm (pH range, 3.0–10.0). The remaining plasma was used for validation. The specimens were then transported to Xining for the following biochemical assays. The study was approved by the ethics committee of Qinghai University School of Medicine (Xining, China).

### 2-DE materials

IPG strips (pH range, 3.0–10.0) were obtained from Bio-Rad (Hercules, CA, USA). Mineral oil, urea, bromophenol blue, 3-[(3-cholamidopropyl)dimethylammonio]-1-propanesulfonate (CHAPS), agarose, acrylamide, Bis, Tris, sodium dodecyl sulfate (SDS), dithiothreitol (DTT), ammonium persulfate, iodoacetamide, tetramethylethylenediamine, sodium thiosulfate, sodium carbonate, potassium ferricyanide, and Trypsin Singles™ proteomics grade were obtained from Sigma-Aldrich (St. Louis, MO, USA). The electrophoresis apparatus, scanner and image analysis software were obtained from Amersham Biosciences (Uppsala, Sweden). Additional analytical-grade chemicals used in this study were from domestic sources. All buffers were prepared with Milli-Q deionized water.

### Depletion of high-abundance plasma proteins

The albumin and immunoglobulin (Ig)G proteins were removed using the Aurum Serum Protein Mini kit (Bio-Rad) to enrich the lower abundance proteins according to the manufacturer’s instructions (20). The protein content of all the samples was determined by Pierce BCA Protein Assay kit (Thermo Fisher Scientific, Waltham, MA, USA).

### 2-DE and mass chromatographic analysis

A volume of each sample that contained 900 μg of total plasma protein based on the BCA results was diluted in rehydration solution containing 7 M urea, 2 M thiourea, 2%(w/v) CHAPS, 0.3% (w/v) DTT and 0.5% (v/v) IPG buffer to a final volume of 450 μl, and applied to each electrophoresis strip. The IPG strips were rehydrated in protein solution for ~15 h under low viscosity paraffin oil (sample loading by rehydration). The IPG strips were then subjected to 1D isoelectric focusing using a flat bed electrophoresis system (IPGphor II; Amersham Biosciences) at room temperature for 17 h to a total of ~80,000 Vh. Upon completion of the first dimension, reduction was performed with DTT (6 M urea, 2% SDS, 0.375 M Tris HCl (pH 8.8), 20% glycerol and 0.2 g DTT) at room temperature for 12 min. A second equilibration step was performed for 12 min in a similar solution, with the exception that DTT was replaced by 2.5% w/v iodoacetamide. The two aforementioned steps were performed with gentle shaking. The second dimension of SDS-polyacrylamide gel electrophoresis was performed on 12.0% homogeneous running gels. The program was as follows: 1 W for 1 h, 2 W for 1 h followed by 48 W until the dye front reached the end of the gel. During this procedure the circulating water temperature was maintained at 12°C.

After the 2-DE procedure was performed, the gels were soaked in a fixing solution containing 40% methanol and 10% acetic acid overnight, and then rinsed in Millipore purified water three times for a total of 60 min. The gels were placed in a Coomassie brilliant blue G-250 solution consisting of 0.12% G-250, 10% (NH_4_)_2_SO_4_, 10% H_3_PO_4_ and 20% methanol (21). All steps were performed with gentle shaking. When protein spots became visible, images of the gels were captured by Umax and LabScan scanners, and analyzed with ImageMaster 7.0 software (22). Automatic spot detection and matching of the gels was performed, followed by manual rechecking of the matched and unmatched protein spots. The intensity volumes of the individual spots were normalized with the total intensity volume of all the spots present in each gel (%V). Differences of >1.5 in expression (ratio, %V) between matched spots were considered significant whenever a spot group passed statistical analysis (t-test, P<0.05) and a second manual verification of the spots on the gel images. All of the protein spots from each individual peptide mass fingerprinting were acquired by Bruker autoflex™ TOF/TOF II (Bruker Corporation, Billerica, MA, USA) after gel digestion. The resulting data combined with molecular weight (Mw) and isoelectric point (pI) was then searched against an online protein database (Mascot; http://www.matrixscience.com) for the identification of the proteins; the score was −10^*^Log_10_(P), where P is the probability that the observed match is a random event. Protein scores of >66 for humans were significant (P<0.05); the proteins were identified and an initial analysis was made accordingly (23).

### Quantitative validation by enzyme-linked immunosorbent assay (ELISA)

The protein quantifications of apolipoprotein A-I (Apo A-I) and Apo A-IV were validated by ELISA with 60 plasma samples, including 20 from patients with HAPE (acute stage and recovery phase), 20 from HAPE-r individuals and 20 from lowland healthy controls according to the manufacturer’s instructions (E0604h and E1967h; USCN Life Science Inc. Wuhan, China)

### Statistical analysis

All data are presented as the mean ± standard deviation. Statistical analysis was performed by one-way analysis of variance followed by a least significant difference post hoc test. P<0.05 was considered to indicate a statistically significant difference.

## Results

### Plasma proteome profiles of patients with HAPE

**Figure 1:**
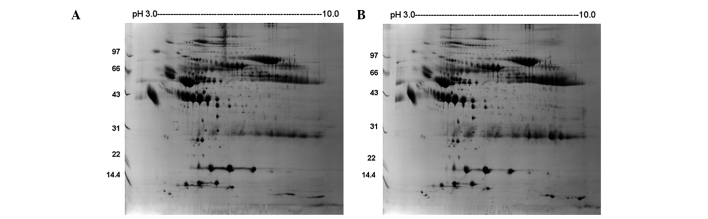
2-DE maps of plasma from one patient with HAPE under the (A) acute and (B) recovery phases. 2-DE, two-dimensional gel elctrophoresis; HAPE, high-altitude pulmonary edema.

**Figure 2:**
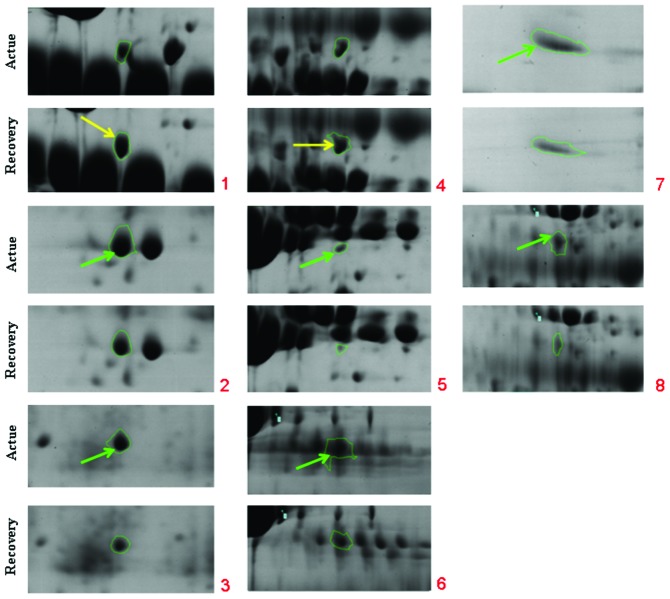
Enlarged regions from 2-DE profiles. Distribution of protein spots with changing expression levels (marked by numbers) and each spot number relates to the data shown in Table I. 2-DE, two-dimensional gel electrophoresis.

**Figure 3:**
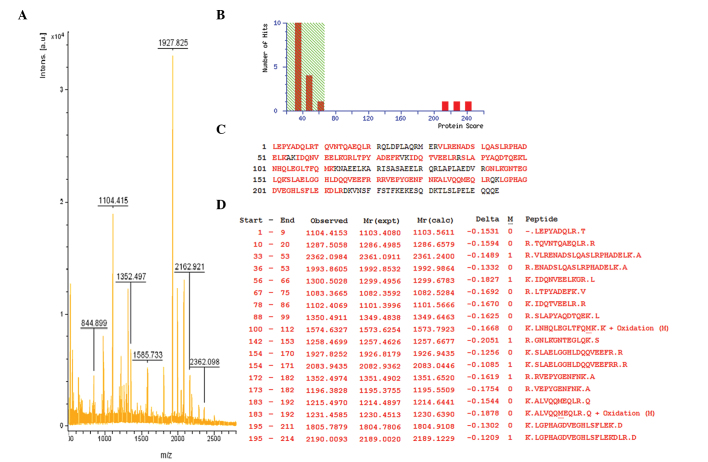
Matrix assisted laser desorption/ionization time of flight mass spectrometry peptide mass fingerprint map and database query result of protein spot 1, which was identified as human apolipoprotein A-IV. (A) MS map of spot 1 in which matched peptide peaks are labeled with mass value. (B) Peptide mass fingerprint was positively matched (score 241) with apolipoprotein A-IV and the probability of a random match was <0.05. (C) NCBI BLAST result, (matched peptide sequences shown in bold red); the observed peptides were highly accorded with apolipoprotein A-IV. (D) Given the mass analysis result between observed peptide sequence and apolipoprotein A-IV sequence, start-end represents the amino acid locus in the protein sequence; observed represents the observed mass; Mr (expt) represents the expected mass; Mr (calc) represents the calculated theoretical mass.

Fig. 1 shows the representative plasma proteome profile of a patient with HAPE. The differences in protein profiles in patients with HAPE at the acute stage and recovery phase were examined using 2-DE with linear IPG strips. More than 300 protein spots in each gel were visualized by the 2D ImageMaster software. The relative intensities of the protein spots (normalized spot volume) were compared and analyzed using the 2-DE gel analysis software. Comparison of the 2-DE results from the patients between the acute and recovery phases led to the selection of eight spots that significantly varied by >1.5-fold in expression level (Table I, Fig. 2). The spots were analyzed using Bruker autoflex TOF/TOF II mass spectrometry. Identification was based on NCBInr, MSDB and SwissProt database entries with the Mascot search engine. Fig. 3 shows the mass spectrometry peptide mass fingerprint map and database query result of protein spot 1, which was identified as human Apo A-IV. Table I lists the SwissProt accession numbers as well as the full names of the protein spots, Mw and pI values, and the percentage of matching peptide and protein amino acid sequence coverage by matching peptides. A comparison of 2-DE gels from patients at different phases indicated that six spots were significantly upregulated in the acute stage and two spots were expressed at higher levels in the recovery phase. Among these changed protein spots, Apo A-I and Apo A-IV were selected for further analysis.

### Validation of Apo A-I and Apo A-IV with ELISA

To validate the results of the proteomic analysis, Apo A-I and Apo A-IV were selected for ELISA (Table II). The mean plasma Apo A-I concentration was 498.3±20.8 μg/ml (mean ± standard deviation) in patients with HAPE at the acute stage versus 430.3±16.0 μg/ml at the recovery phase (P<0.05). The mean Apo A-I concentration was 584.2±60.6 μg/ml in the HAPE-r group versus 436.2±24.9 μg/ml in the lowland controls (P<0.05). The expression level of Apo A-I was upregulated in the plasma of the HAPE-r group and patients with HAPE at the acute stage. The mean plasma Apo A-IV concentration was 14.96±0.43 μg/ml in patients with HAPE at the acute stage versus 16.43±0.76 μg/ml at the recovery phase (P<0.05); and the mean Apo A-IV concentration was 15.70±0.80 μg/ml in HAPE-r versus 21.56±1.80 μg/ml in the lowland controls (P<0.05). The expression levels of Apo A-IV were downregulated in the plasma of the patients with HAPE and the HAPE-r individuals.

## Discussion

Plasma is a primary clinical specimen that is useful for disease diagnosis and therapeutic monitoring (14). The plasma proteome is the most complex human-derived proteome, containing all tissue proteomes as subsets plus numerous distinct immunoglobulins. It has an large dynamic range with >10 orders of magnitude in concentration separating albumin and the rarest proteins, which are now measured clinically or researched in laboratories. With the availability of proteomic tools, the profiling of the human plasma proteome has become increasingly feasible; when searching for disease-related markers, the presence of a particular protein and/or its isoforms in the plasma represents the likelihood of other biologically active molecules as potential biomarkers for disease diagnosis and therapeutic monitoring (24). Cellular functions and the protein expression pattern often change during different disease states. To the best of our knowledge, no previous studies have compared the plasma proteome profile in the acute stage with that of the recovery phase in patients with HAPE. Therefore, the present study aimed to demonstrate the differentially expressed proteins in the plasma of patients with HAPE at different phases, as these have the potential to be developed as biomarkers for the prediction of disease. These differentially expressed proteins may also be important in disease development and recovery.

HAPE usually occurs at altitudes of >3,000 m above sea level in rapidly ascending non-acclimatized individuals (1,2). HAPE is a noncardiogenic pulmonary edema in pulmonary interstitial tissue and alveoli (3). Although hypoxia is a major trigger factor, the pathogenesis of HAPE remains unclear. The search for biomarkers for early-stage prognosis is ongoing. Ren *et al* (25) found out that the plasma concentrations of D-dimer, fibrinogen, fibrin/fibrinogen degradation products (FDP), tissue plasminogen activator (t-PA) and plasminogen activator inhibitor-1 (PAI-1) were significantly higher in patients with HAPE than in the controls and these abnormalities were correlated with the severity of HAPE. Following recovery from HAPE, the plasma concentrations of D-dimer and fibrinogen recovered to normal but the t-PA, PAI-1 and FDP levels in patients with HAPE remained significantly increased compared with those of unacclimatized controls. The development of HAPE is associated with abnormalities in the fibrinolysis and coagulation system, and these abnormalities are correlated with the severity of HAPE. Ahmad *et al* (15) identified 25 protein spots in human plasma of which 14 showed changes in patients with HAPE; these were mainly acute phase proteins, complement components and apolipoproteins. Haptoglobin and Apo A-I were upregulated in the plasma of patients with HAPE.

In the present study, 2-DE followed by mass spectrometry was used to analyze the plasma of patients with HAPE at the acute stage and recovery phase. By comparison of the results of 2-DE from the patients at different phases, eight spots that significantly varied in expression by >1.5-fold were selected; six spots (Apo A-I, antithrombin-III, tubulin β-1 chain, fibrinogen, inter-α inhibitor H3 and serpin peptidase inhibitor) were significantly upregulated in the acute stage and two spots (Apo A-IV and serum amyloid P component) were expressed at higher levels in the recovery phase. Among these changed protein spots, Apo A-I and Apo A-IV were selected for further analysis in the patient and control groups. The Apo A-I concentration was upregulated in patients with HAPE in the acute stage, but was lower compared with that of the HAPE-r group (P<0.05). The Apo A-IV concentrations were downregulated in the plasma of patients with HAPE in the acute stage and the HAPE-r individuals; however, in the recovery phase the Apo A-IV levels were slightly higher in the patients with HAPE than in the HAPE-r individuals (P<0.05). These results are partially supported by those of Ahmad *et al* (15).

The levels of high-density lipoprotein and its major (70%) protein component, Apo A-I, are strongly inversely correlated with the risk of atherosclerosis and other vascular diseases. Apo A-I may contribute to the protective effects, including removal of cholesterol from peripheral tissues to the liver (reverse cholesterol transport), anti-inflammatory and anti-oxidative activities, and modulation of vascular function (15,26). A series of studies have shown that Apo A-I is able to bind LPS to subsequently interrupt the activation of macrophages, inhibit the LPS-activated release of inflammatory cytokines by macrophages and inhibit the activation of neutrophils (27–29), and that Apo A-I overexpression has a protective effect on LPS-induced multiple organ injury (30). Apo A-I has also been shown to be essential for maintaining normal lipid composition and architecture of the lung as well as respiratory physiology (31). In addition, Apo A-I levels were observed to be lower in patients with homozygous sickle cell anemia with pulmonary arterial hypertension (PAH) than in patients with sickle cell anemia without PAH (32). There is emerging evidence that Apo A-I has a critical role in protecting pulmonary artery and airway function as well as preventing inflammation and collagen deposition in the lung (33). Local treatment with Apo A-I is very effective against the development of experimental lung injury and fibrosis (34). Intermittent hypoxic exercise has been shown to stimulate the levels of Apo A-I and strengthen the metabolism of lipids, and may have certain functions in cardiovascular disease treatment (35). In the present study, Apo A-I was found to be upregulated in patients with HAPE (acute stage), but was expressed at lower levels compared with those of the highland controls, suggesting the Apo A-I’s anti-inflammatory and vessel endothelia-protective properties were not enough in HAPE. Therefore, it may be concluded that Apo A-I has an important role in HAPE pathophysiology and may serve as a protective factor for the lung.

Apo A-IV is a 46-kDa glycoprotein. The synthesis and secretion of Apo A-IV is stimulated by fat absorption by intestinal enterocytes, which are incorporated into the surface of nascent chylomicrons, and are important in intestinal lipid absorption and chylomicron assembly (36). Fujimoto demonstrated that Apo A-IV is a satiety signal secreted by the small intestine following the ingestion of a lipid meal (37). Apo A-IV is also present in the hypothalamus, and hypothalamic Apo A-IV levels are reduced by food deprivation and restored by lipid feeding. Apo A-IV is involved in the long-term regulation of food intake and body weight. Chronic ingestion of high fat blunts the hypothalamic Apo A-IV response to lipid feeding and may explain why chronic intake of high fat predisposes animals and humans to obesity (38). Recently, low plasma Apo A-IV levels have been associated with acute coronary syndrome and plasma Apo A-IV levels have been proposed as a potential treatment target for patients with acute coronary syndrome (39). Guo *et al* (40) found Apo A-IV was correlated with the severity of obstructive sleep apnea-hypopnea syndrome. Considering that the expression of Apo A-I and Apo A-IV show variances in HAPE, we hypothesize that these two proteins may become biomarkers for the diagnosis and prognosis of HAPE.

Our future studies will collect more samples, set more than two stages, focus on not only 8 selected proteins, but also the differences of the protein in 2D gel, to illustrate those differential proteins in dynamic change. Due to the importance of correlation study between SNPs in Apo A-I & IV and HAPE, we will select and genotype SNPs, and even whole genes re-sequencing, aiming to illustrate this disease on gene level.

One important limitation of the present study is that it was based on only three patients. In addition, all protein differences in the 2D gel were not estimated; only those spots with significant differences (>1.5-fold) in expression were analyzed and this may omit important information.

## Figures and Tables

**Table I:** Identification of the selected proteins in plasma 2-DE profiles from patients with HAPE.

						Expression		
						
ID	Protein name	Accession no.	Protein score	Sequence coverage (%)	Experimental Mw, (ku)/pI	Acute	Recovery	Fold
1	Apolipoprotein A-IV	AAB59516	241	68	28141/5.39		U	2.0
2	Apolipoprotein A-I	2A01_A	183	51	28061/5.27	U		1.5
3	Serum amyloid P component	1SAC_A	111	28	23358/6.12	U		2.0
4	Antithrombin-III	2B4X_I	108	41	48751/5.72		U	1.5
5	Tubulin β-1 chain	NP_110400	104	34	50865/5.05	U		2.0
6	Fibrinogen	3GHG_A	89	22	61305/7.31	U		2.0
7	Inter-α (globulin) inhibitor H3	BAD96477	84	17	100024/5.45	U		2.0
8	Serpin peptidase inhibitor	EAW90578	77	19	57358/6.47	U		2.0

2-DE, two-dimensional gel electrophoresis; HAPE, high-altitude pulmonary edema; Mw, molecular weight: pI, isoelectric point. U, upregulated.

**Table II:** Comparison of Apo A-I and Apo A-IV concentrations in plasma among HAPE, HAPE-r and the lowland control groups.

Group	No.	Apo A-I (μg/ml)	Apo A-IV (μg/ml)
Acute stage	10	498.3±20.8^a^	14.96±0.43^a^
Recovery phase	10	430.3±16.0^b^	16.43±0.76^a^,^b^
HAPE-r	20	584.2±60.6^a^,^b^	15.70±0.80^a^,^b^
Lowland control	20	436.2±24.9	21.56±1.80

a P<0.05 compared with the lowland control group,

b P<0.05 compared with the acute stage,

c P<0.05 compared with the lowland control group,

Apo, apolipoprotein; HAPE, high-altitude pulmonary edema; HAPE-r, HAPE resistant.
